# Influence of Cardiorespiratory Clinical Placements on the Specialty Interest of Physiotherapy Students

**DOI:** 10.3390/healthcare7040148

**Published:** 2019-11-17

**Authors:** Irene Torres Sánchez, Laura López López, Janet Rodríguez Torres, Esther Prados Román, María Granados Santiago, Marie Carmen Valenza

**Affiliations:** Physical Therapy Department, Faculty of Health Sciences, University of Granada, 18016 Granada, Spain; irenetorres@ugr.es (I.T.S.); lauralopez@ugr.es (L.L.L.); jeanette92@ugr.es (J.R.T.); espra66@hotmail.com (E.P.R.); dulce.maria.1994.25@gmail.com (M.G.S.)

**Keywords:** clinical placements, physiotherapy, specialty interest

## Abstract

Clinical placements are an important part of health students’ training. Whilst much value is placed on the clinical environment as a place to learn, there is a paucity of direct evidence about its effectiveness. The aim of this study was to compare the competence, importance, and interest in cardiorespiratory physiotherapy of students before and after one month of clinical practice. A pre- and post-placement questionnaire about students’ interest in different physiotherapy subspecialties was used. The students with a cardiorespiratory clinical placement showed a significant change in their perception about the importance of the cardiorespiratory specialty (0.348 ± 1.01; *p* < 0.001), while no significant change was observed in the students without cardiorespiratory placement (−0.014 ± 0.825; *p* = 0.883). The presence or absence of clinical placements seems to have a definitive impact on students’ choice of a specialty. This implies the need for developing a set of clinical placements in all the subareas of physiotherapy in order to give undergraduate students the opportunity to make a better decision.

## 1. Introduction

Recently, there has been growing interest in the factors that influence the career and specialty choices of medical [1,2,3,4] and allied health science professionals [5,6,7]. Whilst much value is placed on the clinical environment as a place to learn, there is a paucity of direct evidence about its effectiveness. The clinical placement is considered to be a crucial part of health professionals’ undergraduate education [8,9,10,11], spanning across a range of clinical settings with a variety of qualified clinical supervisors. Learning in clinical settings provides students the opportunity to integrate their theoretical knowledge with practical and professional skills [12].

The clinical setting is defined as an environment that is publicly available for health care purposes and is typically a hospital (both inpatient and outpatient/ambulatory settings), a general practice surgery or a community health care setting. The delivery setting of clinical training within the fields of medicine and nursing has historically been on wards within teaching hospitals. Additional disciplines such as physical therapy, occupational therapy, and social work, for example, have had part of their clinical training in a hospital setting. Training in these and other settings provides an opportunity for students to not only interact with patients but to also be exposed to aspects of their future working environment.

During each clinical placement, physiotherapy students pass through different specialties. A physiotherapy specialty is a prescribed area of physiotherapy practice within which it is possible for a physiotherapist to develop and demonstrate higher levels of knowledge and skills [13]. While the advantages of specialization are clear, the practicality of formalizing the process for specialization of physiotherapy practice is still under debate [14,15]. Previous studies have shown that the physiotherapy specialties that are preferred by students are musculoskeletal, sport, or neurology [16]. However, a lack of interest has been shown for the cardiorespiratory physiotherapy specialty [17]. 

In recent years, various studies have examined the attitudinal factors associated with students’ decision to pursue a specialty in different medical subspecialties. These studies indicate that students tend to be considerably influenced by perceived career rewards, such as income [18,19,20], prestige [13,18,19], job opportunities [13,18,19], and career satisfaction [21], when they choose a specialty. In physiotherapy, the main factors influencing these choices were reported as being influenced by educators/clinicians, being increasingly exposed to various physiotherapy specialties through clinical practice, and increasing clinical experience and competencies in certain specialties [17]. In particular, the interest in physiotherapy cardiorespiratory specialty has been shown to be influenced by an individual’s own interest in the area, the opportunity to work with patients in an acute respiratory setting, and the role of educators [22]. However, the repercussion of clinical practice in cardiorespiratory settings on the students’ choice has not been evaluated.

The aim of this study was to compare the competence, importance, and interest in cardiorespiratory physiotherapy of students before and after one month of clinical practice. 

## 2. Materials and Methods 

A prospective study was conducted in three schools of physiotherapy in Spain located in Las Palmas de Gran Canaria, Toledo, and Granada. As they were well distributed geographically, the results can be considered to be representative of the whole country. To be included in the study, participants had to be third-year physiotherapy students at one of the three universities. Students with previous clinical cardiorespiratory placement or experiences were excluded.

All subjects gave their written informed consent for inclusion before they participated in the study. Students were advised that participation in the study was not required, it was anonymous, and participation would not impact their grades. The study was conducted in accordance with the Declaration of Helsinki, and the protocol of the study was approved by the Institutional Review Board of the University. The Strengthening the. Reporting of Observational studies in Epidemiology (STROBE) guideline was followed during the course of the research [23].

Students completed a questionnaire two times; a questionnaire was administered before and after completion of one month of clinical placements. The questionnaire used was developed on the basis of a previous similar study [24] and included closed-ended items and adjectival/adverbial rating scales. The students were asked to assign a value using a Likert scale, where 0 is “no importance” and 5 is “the most important”, their perceptions about their feelings and their competence in regard to different physiotherapy subspecialties. Items included in the questionnaire are presented below.

Importance of cardiorespiratory: 0 (no importance)–5 (the most important)
Intention of specializing in cardiorespiratory: Yes/No/I don’t know
Perceived competence in cardiorespiratory: 0 (no importance)–5 (the most important)
Perceived competence in cardiorespiratory relative to other specialties: More than other specialties/Less than other specialties/The same

Values assigned to various clinical areas of physiotherapy:

Cardiorespiratory: 0 (no importance)–5 (the most important)
Musculoskeletal physiotherapy: 0 (no importance)–5 (the most important)
Neurological physiotherapy: 0 (no importance)–5 (the most important)
Geriatric physiotherapy: 0 (no importance)–5 (the most important)
Mental health physiotherapy: 0 (no importance)–5 (the most important)
Community physiotherapy: 0 (no importance)–5 (the most important)

After received clinical placements, students were divided into two groups: one group of students with cardiorespiratory clinical placement, and one group of students without cardiorespiratory clinical placement.

### Statistical Analysis

The data were analyzed with the Statistical Package for Social Sciences (SPSS) software version 20.0 (IBM, Armonk, NY, USA). Absolute and relative frequencies of categorical variables were calculated. For continuous variables, central tendency and dispersion measures were determined. Differences between pre- and post-cardiorespiratory clinical placements were compared with a Student *t*-test for continuous data and χ2 tests of independence for categorical data. The statistical analysis was conducted at 95% confidence level. A *p*-value of less than 0.05 was considered statistically significant.

## 3. Results

### 3.1. Distribution and Returning of Questionnaires

**Figure 1:**
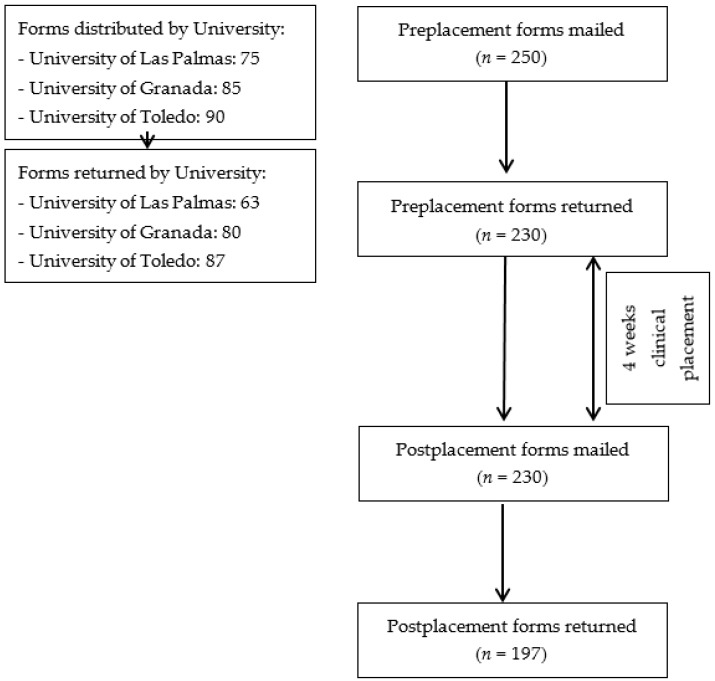
Distribution and returning of questionnaires.

Two hundred and fifty forms were distributed at three schools of physiotherapy in Spain (Las Palmas de Gran Canaria, Toledo, and Granada). Reminders were sent to maximize the return rate. Two hundred and thirty forms were returned for the participants’ pre-clinical placement interest in cardiorespiratory specialty. After one month of clinical placements, the participants were asked to complete the same survey in order to indicate their post-clinical placement interest in cardiorespiratory specialty. Some of the participants did not return the pre-placement or post-placement forms and were lost in the follow up. Finally, 78.8% (*n* = 197) of forms were returned. The questionnaires were collected anonymously. The distribution and return rate of questionnaires are presented in Figure 1.

### 3.2. Characteristics of the Students

The characteristics of the students are shown in Table 1.

A minority of students (37.05%, *n* = 73) had a cardiorespiratory placement, while 62.94% (*n* = 124) had other placements. 

The most frequently reported preferred workplace of students was private practice, while nursing homes were the least prevalent work choice of students (48.3%, 43.9%, and 53.12% vs. 15.6%, 18.5%, and 21.6%).

### 3.3. Importance, Competence, and Interest in Cardiorespiratory Specialty

Opinions and values of cardiorespiratory and other physiotherapy specialties are reported in Table 2. The values were obtained prior to the clinical placement development. 

Data are expressed as means ± standard deviation for continuous variables and percentages for categorical data.

The importance attributed to cardiorespiratory specialty was 4.69 ± 0.626, where 1 is the worst value and 5 is the highest value. 

When students were asked about their intention to specialize in cardiorespiratory, “I don’t know” was the answer reported most frequently (61.8%). 

Musculoskeletal physiotherapy was the area that received the highest value, while mental health physiotherapy was the received the lowest value.

### 3.4. Changes between Pre-Placements and Post-Placements

Changes in opinions and values of cardiorespiratory specialty after the clinical placement, by group, can be observed in Table 3.

While most of the explored values showed significant changes (*p* < 0.05) in the cardiorespiratory placement group, only the perceived competence in cardiorespiratory and the intention to specialize in cardiorespiratory showed significant changes in the students without cardiorespiratory placements.

Curiously, perceived competence in cardiorespiratory showed no change in any group (*p* = 0.901 and *p* = 0.826).

Significant differences were found between the pre-placement and post-placement values assigned to cardiorespiratory, neurological, geriatric, and community physiotherapy by the students with a cardiorespiratory placement (*p* < 0.05). 

## 4. Discussion

The aim of this study was to compare the competence, importance, and interest in cardiorespiratory physiotherapy of students before and after one month of clinical practice. Although interest in cardiorespiratory as a specialty has already been studied [17], the effect of clinical experience on this decision has never been taken into account before. Studies undertaken in several countries [6,24,25] have shown a lack of awareness of cardiorespiratory on entry to physiotherapy undergraduate programs and a lack of interest in specializing in cardiorespiratory on completion of undergraduate programs. The present study found that specific cardiorespiratory clinical placements positively influence students’ interest in cardiorespiratory specialty.

Problems in recruitment into specialist cardiorespiratory positions in some countries have been found [26]. Dodson, et al. [27] surveyed the career aspirations of final (fourth) year physiotherapists in New Zealand, focusing specifically on areas in which students intended to seek employment following qualification. The main career goal of the students was to own a private practice primarily focusing on sports physiotherapy. Other studies [6] have also reported that physiotherapy students would prefer to have future careers in sports medicine clinics and fitness centers; additionally, the idea of setting up a private practice has been shown to be highly endorsed by a majority of students [28]. Similarly, to these research studies, the present study found that most students wished to specialize in musculoskeletal physiotherapy and neurology, while cardiorespiratory was one of the lowest ranked choices of physiotherapy career specialization. 

Owen and colleagues [29] found that the determinants of students’ specialty choice are multifactorial and dynamic. Studies conducted on medical undergraduate students have found that students’ clinical encounters with patients are an essential component of undergraduate clinical education [24,30], finding changes in the perception and acquisition of clinical abilities. The study conducted by Bennett and Hartberg [31] suggested that students undergoing a cardiorespiratory clinical placement were more likely to specialize in cardiorespiratory. Our study confirms the same idea, using a control group that did not have a cardiorespiratory placement, as students found the cardiorespiratory specialty to be more important after having a clinical placement in this area. 

The repercussion of clinical placements on health students’ interests seems to have a definitive impact in their chosen of a specialty. This highlights the importance of having undergraduate students experience clinical placement in all the subareas of physiotherapy in order to give students the opportunity to make a better decision. Including at least one specific clinical placement in cardiorespiratory settings during the students’ program may increase awareness about the importance of cardiorespiratory physiotherapy specialty. 

Clinical placement may increase interest in an area that is different from the specialty of the placement, however, the interest in the area of the clinical placement may be greater. In a previous study, it was shown that in nursing the majority of participants applied for first destination posts in areas in which they had been placed as a student [32].

A previous study also found that a positive clinical placement had the greatest influence on students’ self-reported knowledge, skills, attitudes, and interest; however, the quantity of theoretical education also emerged as an influencing variable [33]. 

Future studies could examine other factors apart from clinical placement that could influence students’ choice.

The main limitation of this study is the use of a non-validated questionnaire; however, it was developed on the basis of a previous similar study [24]. Another limitation is the sample of participants, who were recruited from three universities: Las Palmas de Gran Canaria, Toledo. and Granada. However, as they were well distributed geographically, the results could be considered to be representative of the whole country.

## 5. Conclusions

Clinical placements seem to influence awareness of students about the importance of physiotherapy specialties. Perceptions of clinical educators may provide key information about the clinical perception of cardiorespiratory specialty that can be used to develop more effective strategies to increase interest in cardiorespiratory specialty.

## Figures and Tables

**Table 1:** Sample description according to cardiorespiratory-related clinical placement.

Variables	Students with Cardiorespiratory Clinical Placement (*n* = 73)	Students without Cardiorespiratory Clinical Placement (*n* = 124)	*p*-Value
**School participation rate:**			
Las Palmas (%)	20	37	
Toledo (%)	22	35	0.178
Granada (%)	31	51	
**Gender**			
% of males	31.2	23.8	0.009 **
Age (years)	23.25 ± 2.1	23.62 ± 1.85	0.652
**Preferred workplace (%)**			
Private practice	48.3	43.9	
Hospital	35.1	35.2	
Nursing home	15.6	18.5	
Other	1	2.4	<0.001 **
**Probable workplace (%)**			
Private practice	46.3	44.8	
Hospital	2	5	
Nursing home	47.1	43	
Other	4.6	7.2	<0.001 **

Data are expressed as means ± standard deviation for continuous variables and percentages for categorical data. **, *p* < 0.001.

**Table 2:** Values assigned to competence, importance, and interest in cardiorespiratory physiotherapy of all the students included in the study.

Variables	Students Sample (*n* = 197)	Range
Importance of cardiorespiratory	4.69 ± 0.626	[2,3,4,5]
Intention of specializing in cardiorespiratory (%)		
Yes	11	
No	27.2	
I don’t know	61.8	
Perceived competence in cardiorespiratory	2.92 ± 1.09	[1,2,3,4,5]
**Perceived competence in cardiorespiratory relative to other specialties:**		
More than other specialties	10.8	
Less than other specialties	40.5	
The same	48.7	
**Values assigned to various clinical areas of physiotherapy:**		
Cardiorespiratory	3.87 ± 1.138	[1,2,3,4,5]
Musculoskeletal physiotherapy	4.75 ± 0.608	[2,3,4,5]
Neurological physiotherapy	4.51 ± 0.802	[1,2,3,4,5]
Geriatric physiotherapy	3.87 ± 1.138	[1,2,3,4,5]
Mental health physiotherapy	3.23 ± 0.983	[1,2,3,4,5]
Community physiotherapy	3.35 ± 0.920	[1,2,3,4,5]

**Table 3:** Changes between pre-placement and post-placement values assigned to competence, importance, and interest in cardiorespiratory physiotherapy of all the students included in the study by group.

Variables	Students with Cardiorespiratory Placement (*n* = 73)		Students without Cardiorespiratory Placement (*n* = 124)	
	Average Change	*p*-Value	Average Change	*p*-Value
Importance of cardiorespiratory	0.348 ± 1.01	*p* < 0.001 **	−0.014 ± 0.825	0.883
Intention of specializing in cardiorespiratory	% change		% change	
Yes				
No	−8.8		−7.3	
I don’t know	−0.2	*p* < 0.001 **	1.4	*p* < 0.001 **
	8.5		−6.3	
Perceived competence in cardiorespiratory	0.017 ± 1.5	0.901	−0.044 ± 1.66	0.826
**Perceived competence in cardiorespiratory relative to other specialties:**				
More than other specialties	−7.7	*p* < 0.001 **	−3.2	*p* < 0.001 **
Less than other specialties	−0.7		−3.1	
The same	8.3		6.3	
**Values assigned to various clinical areas of physiotherapy:**				
Cardiorespiratory	0.398 ± 1.552	0.009 *	−0.175 ± 1.95	0.946
Musculoskeletal physiotherapy	0.111 ± 0.99	0.252	0.087 ± 0.575	0.255
Neurological physiotherapy	0.314 ± 1.257	0.011 *	0.157 ± 1.06	0.268
Geriatric physiotherapy	0.252 ± 1.289	0.045 *	0.017 ± 1.15	0.909
Mental health physiotherapy	0.214 ± 1.37	0.109	0.107 ± 1.18	0.502
Community physiotherapy	0.264 ± 1.34	0.045 *	0.14 ± 1.0	0.298

Data are expressed as means differences ± standard deviation for continuous variables and percentages for categorical data. *, *p* < 0.05; **, *p* < 0.001.
